# A Novel Visual Analysis Method of Food Safety Risk Traceability Based on Blockchain

**DOI:** 10.3390/ijerph17072300

**Published:** 2020-03-29

**Authors:** Zhihao Hao, Dianhui Mao, Bob Zhang, Min Zuo, Zhihua Zhao

**Affiliations:** 1 National Engineering Laboratory for Agri-product Quality Traceability, Beijing Technology and Business University, Beijing 100048, China; hao_zhihao@126.com (Z.H.); zuomin@th.btbu.edu.cn (M.Z.); 2 PAMI Research Group, Department of Computer and Information Science, University of Macau, Taipa, Macau 999078, China; 3 Beijing Key Laboratory of Big Data Technology for Food Safety, School of Computer and Information Engineering, Beijing Technology and Business University, Beijing 100048, China; 4 The School of Law, Chinese University of Political Science and Law, Beijing 102249, China; zhaozhihua@cupl.edu.cn

**Keywords:** blockchain, visualization, risk, traceability, food safety

## Abstract

Current food traceability systems have a number of problems, such as data being easily tampered with and a lack of effective methods to intuitively analyze the causes of risks. Therefore, a novel method has been proposed that combines blockchain technology with visualization technology, which uses Hyperledger to build an information storage platform. Features such as distribution and tamper-resistance can guarantee the authenticity and validity of data. A data structure model is designed to implement the data storage of the blockchain. The food safety risks of unqualified detection data can be quantitatively analyzed, and a food safety risk assessment model is established according to failure rate and qualification deviation. Risk analysis used visual techniques, such as heat maps, to show the areas where unqualified products appeared, with a migration map and a force-directed graph used to trace these products. Moreover, the food sampling data were used as the experimental data set to test the validity of the method. Instead of difficult-to-understand and highly specialized food data sets, such as elements in food, food sampling data for the entire year of 2016 was used to analyze the risks of food incidents. A case study using aquatic products as an example was explored, where the results showed the risks intuitively. Furthermore, by analyzing the reasons and traceability processes effectively, it can be proven that the proposed method provides a basis to formulate a regulatory strategy for regions with risks.

## 1. Introduction

With the improvement of living standards, people have higher requirements for food quality. Ensuring food quality safety has become a major problem for governments, business organizations, and merchants. However, due to the complexity of the food supply chain, there is no effective regulatory mechanism from farm to table, which has led to frequent food safety issues around the world over the past few decades. For example, according to The Sunday Times, on June 5, 2005, the British Food Standards Agency found the carcinogen “malachite green” in salmon sold in a well-known supermarket. Even in recent years, food safety issues have not been well resolved. In 2019, hundreds of African swine fever epidemics have occurred in many provinces of China [1]. These directly caused an increase of pork prices and seriously affected people’s daily lives.

At present, it is difficult to recall food quickly after it has entered the market. This is because the storage of food information is incomplete and the stored information can be easily forged. Countries around the world have adopted different regulatory approaches to prevent food safety incidents. For example, the United States has utilized product traceability systems and product recall systems [2]. The European Union (EU) regulates the production, distribution, and processing of the entire food chain from farm to table [3]. China has used a segmented management safety oversight model [4]. This traditional regulatory model will lead to independent law enforcement by various departments. Each supervisory authority is responsible for its own affairs, or can shirk its responsibilities with other authorities, which is not conducive to supervision. In addition, due to the large number of food safety supervision departments and the lack of effective coordination departments and mechanisms, the regulatory information of a certain product cannot be effectively transmitted to the next stage. This may lead to repeated sampling and waste of manpower and resources. In addition to these issues, there are a number of problems with these models; for example, information can be easily tampered with, sources can be difficult to track, and so on.

In recent years, blockchain technology has received increasing attention due to features such as having a distributed system, decentralization, and generating data that cannot be tampered with [5]. Blockchain is a distributed database system containing multiple independent nodes. It is essentially a distributed ledger that is maintained jointly by the nodes in the network. Information can be recorded into the blockchain to ensure its security and credibility. It implements decentralized point-to-point transactions in distributed networks by using encryption algorithms, timestamps, consensus mechanisms, and reward mechanisms. The process is efficient and transparent. This technology solves the problems of poor reliability, low security, high cost, and low efficiency in the current centralized storage mode.

The introduction of blockchain technology into food safety supervision is becoming a trend. In October 2016, the Wal-Mart Food Safety Cooperation Center, International Business Machines Corporation (IBM), and Tsinghua University piloted a food traceability system based on blockchain technology in China. In March 2017, Alibaba cooperated with PricewaterhouseCoopers to launch a food supply blockchain test project in the food supply chain. In addition, Walmart has required its vegetable suppliers to use blockchain technology developed by IBM to track products in real time since September 2019. These practices have proven to the public that stakeholders in the global food supply chain consider food safety as being collaborative rather than competitive. Blockchain technology provides a new tool for traceability. By applying blockchain technology to food traceability systems, the authenticity of data can be guaranteed to solve trust issues. In addition, establishing a more transparent and traceable food system can ensure that every stakeholder in the blockchain, such as food producers, processors, retailers, and consumers, can benefit from it. For example, Tian [6] has built a traceability system for an agricultural product supply chain based on Radio Frequency Identification (RFID) and blockchain technology, which covers the entire process of data collection and information management in each link of the supply chain and realizes the monitoring, tracking, and traceability management of agricultural food quality and safety. 

However, there are two main challenges. One is the lack of an intuitive display and analysis of the vast amounts of data. Visualization technologies can help people quickly identify and make relevant decisions. For example, Saura et al. [7] extracted useful knowledge from available user generated content (UGC) samples and visualized them. The results obtained are relevant to innovative education trends, and practitioners can use them to improve strategies and interventions in the education sector in the short-term future. Another challenge is that it is difficult to establish a good risk assessment model for food safety risks. Risk assessment refers to the use of the employed method to analyze existing data to assess the possibility of potential risks [8], and is applied to the food industry to evaluate food safety risks. Its basic content includes hazard identification, hazard feature description, exposure assessment, and risk feature description [9]. Therefore, visualization technologies and risk assessment models can be introduced into the food industry, as they help people formulate relevant strategies to reduce the risk of food safety incidents.

In order to meet these challenges, we propose a visual analysis method of food safety risk traceability based on blockchain. The theoretical framework and implementation process of this research were divided into three steps. First, we designed a data structure for users with different identity roles to update, view, and obtain information. Here, users can also upload relevant information to the network for verification. Food sampling data are used in this research for food safety risk assessment, where a method to quantitatively analyze food safety risks is proposed according to the food sampling data to facilitate traceability analysis. After the information is verified, it is uploaded to the blockchain. The consortium blockchain Hyperledger Fabric is used as a data hosting platform. Ordinary users and regulators are given different identity permissions in the system to perform different functions. Finally, visualization technologies are used to analyze food safety risk assessment results and food safety risk traceability processes based on spatial characteristics of the data in the blockchain. Heat maps are used to illustrate macro risks. Migration maps and force-directed graphs are also applied to show microscopic flow. By using a human’s rapid recognition ability in visual mode, people can easily track and monitor the extent and impact of the dangerous spreading of food(s).

The rest of this research is organized as follows. Related work is described in Section 2. Section 3 presents the framework. Design and implementation are illustrated in Section 4. Section 5 shows experiments and analysis, and the conclusion is given in Section 6.

## 2. Relate Work 

Currently, many technologies have been used in the food industry to solve frequent food safety incidents, with one example being RFID (Radio Frequency Identification) [10]. For example, Zhang et al. [11] proposed a special food safety traceability network model based on RFID technology, which applies RFID technology to data acquisition of raw material procurement, production processing, warehousing management, logistics, and transportation. However, traditional models using RFID technology have problems such as low efficiency. In order to solve these problems, Alfian et al. [12] used Internet of Things (IoT) technology and machine learning methods to improve the efficiency of RFID-based perishable food traceability systems. In addition, Fan et al. [13] proposed a method to improve the continuous traceability of food by using barcode RFID two-way conversion equipment. In addition to RFID, IoT technology is also widely used in the food industry [14]. For example, Verdouw et al. [15] developed and applied a framework for the food industry, which is based on the Internet of Things system for modeling. However, these technologies still have some drawbacks. For example, the centralized storage of data increases the possibility of information loss and tampering. In addition, there are other problems, such as low transparency and easy leakage of information. For instance, the largest information leakage incident of South Korea occurred in 2013, where 104 million people’s personal information was leaked [16].

Recently, blockchain technology has been applied to the food industry [17,18]. Tse et al. [19] proposed a method of applying blockchain technology to the food supply chain for ensured information security. Unlike the traditional food supply chain traceability system, it is transformed into a distributed storage platform based on the underlying protocol of the blockchain to ensure data security and traceability [20]. Blockchain technology is often combined with other technologies. For example, Hong et al. [21] implemented a traceability system based on the Internet of Things and blockchain technology. Tsang et al. [22] used the Internet of Things to achieve traceability through integrated consensus mechanisms. Through the application of blockchain, the environment of the food supply chain has been greatly improved [23].

However, current data about the food industry requires professional visualization methods to help quantitative analysis [24,25]. For example, ElMasry et al. [26] used near-infrared hyperspectral imaging to quantitatively analyze the prediction parameters of fresh beef. Cropotova et al. proposed a fluorimetric assay method to quantitatively analyze protein carbonyls [27]. Lohumi et al. [28] used Fourier transform infrared (FTIR) spectroscopy to quantitatively analyze Sudan dye adulteration for risk assessment. However, these results may cause great difficulties to the understanding of ordinary people. Therefore, suitable data sets are needed to help users understand and avoid food safety risks. Therefore, we chose food sampling data as the data set for experimental testing. Blockchain technology is used to ensure the true validity of the data, and visual methods, such as heat maps and migration maps, are used to display and analyze risks.

## 3. Framework 

The method uses Hyperledger Fabric as the underlying technology. Hyperledger is derived from the open source project led by the Linux Foundation in 2015. Hyperledger Fabric is its sub-project, which allows multiple parties to participate in the development, deployment, and operation of the consortium blockchain platform. It aims to create an extensible blockchain development framework that provides solutions for the development of enterprise-level blockchain applications.

In order to better understand Hyperledger Fabric, its architecture is introduced briefly here. Hyperledger Fabric includes multiple components: (1) Orderer. In Hyperledger Fabric, an ordering service is provided through multiple Orderers. They receive all transactions from the entire network and packs the transactions into blocks in order of time. It does not participate in the execution and verification of the transaction, so it does not care about the specific content of the transaction. The goal is to reach a consensus on the order in which the transactions occur, and then broadcast the results. (2) Client. The client is the access point between the user and the Hyperledger Fabric network, and deploys a proprietary Software Development Kit (SDK). Users can use the client to initiate a transaction request. (3) Endorser. When a client wants to initiate a transaction, it must first obtain a certain number of endorsements from Endorsers for the transaction, that is, signing to prove that the transaction has been processed by the endorsing nodes. (4) Committer. This type of node is the main body for maintaining the ledger in the network. Committers can receive packaged blocks and verify the validity of transactions in the blocks, and submit valid transactions to the ledger. Endorsers are special Committers. Their endorsement function is an additional function.

**Figure 1:**
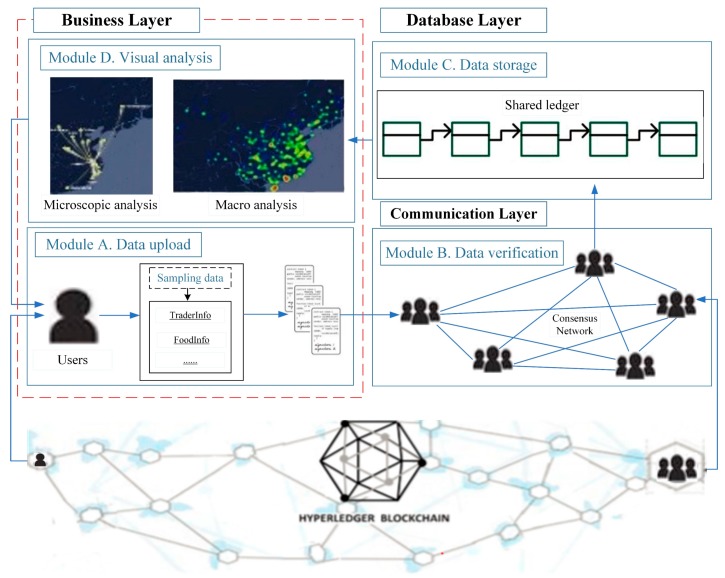
The framework of the method.

The framework is shown in Figure 1 and has three layers as follows.

Business Layer. This layer supports user access and contains entry points for human-computer interaction. It consists of modules A and D in Figure 1. Module A is the operation of uploading data, and Module D is the visual display. Module A is mainly applicable to business developers who deploy smart contracts at the business level. Smart contracts can be understood as script code running on the blockchain, which provides programmable functions to support upper-layer applications. Users write their own smart contracts through the Application Programming Interface (API). Users can update or obtain information in the blockchain through smart contracts. Module D is the information visualization part. The results of this module can be fed back to users to help the user perform risk and traceability analysis. The heat map can show the risks in a macro view. The area shown by the heat map is an important attribute of food risk data and the basis of food safety traceability. Based on this, force-directed graphs and migration maps can be used to show the microscopic flow of products to analyze their traceability. These techniques can produce direct, simple, and high-quality results.

Communication layer. This layer contains the network structure and protocols of the P2P network (Module B). It provides network services for the blockchain platform and uses the Gossip data communication protocol to achieve state synchronization and data distribution between nodes in the network. Due to the use of Hyperledger Fabric, nodes in the communication layer are assigned different roles to execute various services [29]. The communication layer not only deals directly with the business layer, but also connects with the database layer. The uploaded data is verified by the consensus algorithm and then uploaded to the blockchain to achieve consistency and correctness of the ledger data on different ledger nodes. Consensus algorithms are the foundation of blockchain technology. PBFT (Practical Byzantine Fault Tolerance) is used in Hyperledger Fabric. The main steps are: (1) The client (user) sends a request to activate the service operation of the master node (regulator). (2) After receiving the request, the master node broadcasts the request to each node. (3) The client waits for responses from different nodes. If more than half of the nodes (for example, 51%) have the same response, it is the result recorded in the blockchain.

Database layer. As can be seen from Module C, this layer consists of a shared ledger. Here, we create a data structure model to implement different types of data upload. The uploaded information is stored in blocks. Each block consists of a block header and a block body. The block header is divided into a few parts, such as version (the version number of the block header, used to track software/protocol updates), prevBlockHash (the hash address of the previous block), merkleRoot (the hash value of the Merkle tree root in the block), time (the creation timestamp of the block), and so on. The block body contains transaction information, which is the number of transactions and transaction details such as location, time, etc. The information recorded in the ledger is used as data for visual analysis.

## 4. System Design and Implements

### 4.1. Data Structure and Storage Process

**Figure 2:**
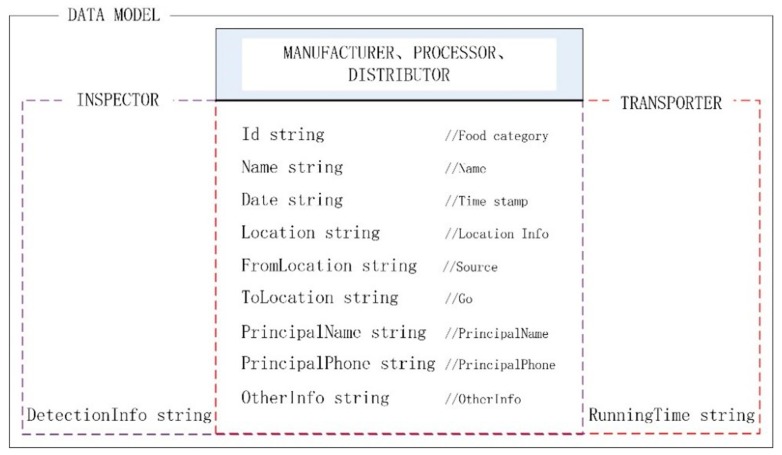
Data structure model for uploading to the blockchain.

A custom data model is created, as shown in Figure 2. The data model includes detailed parameters such as its identification and information for uploading. There are nine fields in total, which are ID, Name, Date, Location, FromLocation, ToLocation, PrincipalName, PrincipalPhone, and OtherInfo. Among these, ID is the classification of food categories, such as: milk and dairy products, fats, oils and emulsified fat productsand cereals. Name refers to the sub-categories of food, such as high calcium milk. Date is the timestamp of data uploaded for each link. *Location* represents the geographical coordinates of the current link. The previous geographical coordinates are defined as FromLocation. ToLocation represents the next geographical coordinates. RunningTime is the transport time. PrincipalName the name of the principal in this link. PrincipalPhone represents phone calls of the principal. OtherInfo is the reserved field. Each link uploads a different type of data, but the ID and *Name* fields must be uploaded. Other fields can be filled out based on the specific link and role. The format of the key-value store sets the data format to JavaScript Object Notation (JSON). Therefore, these data models eventually need to be converted to JSON strings.

In addition, there are five roles for data upload: manufacturer, processor, inspector, transporter, and distributor. Different roles have different responsibilities and parameters. The three roles of manufacturer, processor and distributor can use all of the above parameters. Different from these roles, inspector can use one more parameter, DetectionInfo, and transporter can use the other parameter RunningTime.

**Figure 3:**
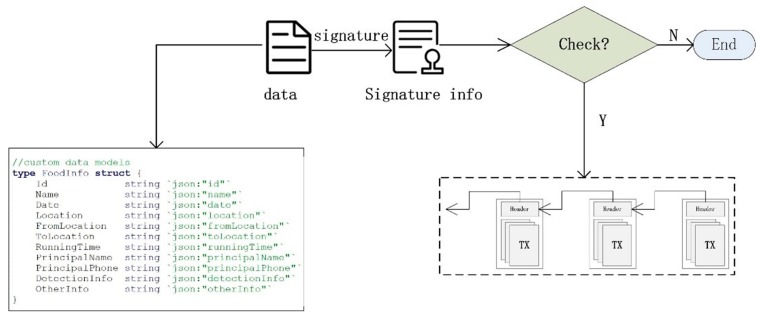
The process of recording data into the blockchain.

The process of data recording to the blockchain is shown in Figure 3. When a participant initiates a request, the system will call an embedded smart contract and then verify the data structure, signature integrity, and whether it is duplicated. After the verification is passed, the custom smart contract will be called to upload the data to the blockchain. Nodes in the blockchain network can access different information through different rights. In addition, digital signatures are used to ensure the integrity of the information. The digital signature in this research uses asymmetric encryption technology. It ensures that the information cannot be tampered with, and the identity information of the two nodes does not need to be disclosed. When the process is completed, the smart contract will automatically execute the content of the agreement. For example, during the transaction, both parties use asymmetric keys to encrypt and decrypt transaction information. It guarantees that the transaction information will not be tampered with maliciously, and solves the integrity problem in the transaction process.

### 4.2. Quantitative Analysis of Safety Risks 

Risk assessment refers to the consideration of all available relevant data, and on this basis to identify areas where risks may arise. The results of food safety risk assessments can provide a basis for regulators to formulate regulatory strategies. When constructing food safety risk indicators, we study the degree of risk of food through the rate of non-conformity and the degree of deviation from conformity. The failure rate describes the frequency with which foods that do not meet the required standards [30] occur (unqualified products). The deviation rate describes how far these unqualified products deviate from their safety standards. They can be calculated using the following procedure. 

The specific detection results of the product *i* in a region are defined as $m_{i}^{p}$, where *p* is the type of product. $m_{i}^{p}$ needs to be compared with [$Min^{p}$, *Max^p^*], which is the scope of safety standards, and unqualified number count $E_{i}^{p}\;\left(E_{i}^{p}\in \;\left[0,1\right]\right)$ can be notified by following:

- If $Min^{p}\;\leq \;m_{i}^{p}\;\leq Max^{p}\;$, then the detected item meets safety standards,$\;E_{i}^{p}\;$= 0.
- If $m_{i}^{p}<Min^{p}$ or $m_{i}^{p}>$. $Max^{p}$, then the detected item does not meet safety standards, $E_{i}^{p}\;$ = 1.

Thus, the failure rate ${\tilde{V}}_{i}^{p}$ can be calculated by:

(1) $${\tilde{V}}_{i}^{p}=\frac{\sum^{n_{i}^{p}}E_{i}^{p}}{n_{i}^{p}}.\;$$

where $n_{i}^{p}$ is the number of sampled products. For unqualified products, the deviation rate needs to be calculated, as shown in Equation (2). It is represented by $B_{i}^{p}$ ($0<B_{i}^{p}\leq 1).$

(2) $$B_{i}^{p}=\{\begin{array}{c} \frac{Min^{p}-m_{i}^{p}}{Min^{p}},\;0<m_{i}^{p}<Min^{p}\\ \frac{\;m_{i}^{p}-Max^{p}\;}{m_{i}^{p}},0<Max^{p}<m_{i}^{p} \end{array}$$

The average deviation rate ${\tilde{B}}_{i}^{p}\;$ can be calculated by:

(3) $${\tilde{B}}_{i}^{p}=\frac{\sum^{n_{i}^{p}}B_{i}^{p}}{n_{i}^{p}}$$

For a region *j*, the risk indicator can be calculated according to this formula:

(4) $$\psi_{j}=\sum_{1}^{n}({\tilde{V}}_{i}^{p}\times {\tilde{B}}_{i}^{p})$$

This obtains the risk indicators set $\left(\psi_{1},\;\psi_{2},\psi_{3},\ldots ,\psi_{n}\right)$. After sorting, the region set with risks can be acquired according to the results in the order from large to small. Then, these can be analyzed using visualization techniques.

### 4.3. Visual Analysis Methods

Visual analysis consists of macro and micro analysis. The macro analysis method uses heat map technology to illustrate the risk distributions of regions. For a specific region, the migration map and force-directed graph technologies are used to demonstrate the reasons why these risks occur. The details of these methods are explained in the following.

#### 4.3.1. Macro Analysis

The macro analysis of risks can be processed by heat maps [31]. These display areas of interest to users in colored highlights. The heat map generation process is roughly divided into three steps. First, the original data needs to be clustered to form clusters. Then, Gaussian fitting is performed according to the center points of the clusters to obtain each heat value in regards to its surrounding area. Finally, the heat map can be formed by coloring according to the heat value, which is combined with maps for intuitive display. The detailed process is as follows.

Clustering data is the basis of heat map generation. Clustering refers to clustering similar entities together to form a cluster [32]. The spatial distance between the data can be used as the basis for clustering. In a cluster obtained by clustering data with spatial-temporal characteristics (spatial points), the distance between any two spatial points must be smaller than the distance between any point in other clusters. From the aspect of density, the tight aggregation of points forms a cluster with high density and a cluster with low density represents points that are scattered. The operation of clustering is summarized as follows.

- 1. Data initialization. Data dimensions are reduced and certain characteristics are standardized.
- 2. Selection of data characteristics. The characteristics that can best distinguish the data are found, extracted, and stored.
- 3. Data clustering. Select or construct a certain clustering function to test the similarity of the data according to the characteristics, and perform clustering based on the test results.
- 4. Data cluster evaluation [33]. Perform evaluation of correlation and validity on clusters.

Processing the original data based on the grid clustering algorithm can preserve the important attributes of the data [34]. The efficiency of a grid cluster will not depend on the amount of raw data. It is determined by the number of elements in a one-dimensional case. It can improve data processing efficiency by flexibly processing the amount of data at different levels. The risk distribution can be shown by heat maps after the above operation of the risk regions (hotspots). These hotspots will produce a certain range of influence, and the strength of this range of influence can be calculated using a Gaussian function, as shown in Equation (5).

(5) $$r\left(x\right)=\frac{k}{\sigma \sqrt{2\pi }}e^{-\frac{x^{2}}{2\sigma^{2}}}$$

where *x* is the distance between sampling site and hotspot, σ is the scale factor of the function, and *k* is the influence factor. The larger of these two factors, the wider the range of influence. Equation (5) shows that its range of action is inversely proportional to distance. The influence of the center area is the largest, and the periphery is the smallest. When processing a certain area, it is necessary to add up all the heat values that can be generated in the surrounding area. Therefore, the final heat value of a certain region can be calculated by:

(6) $$H=\sum_{i=1}^{n}r\left(x\right)$$

After H is obtained, the corresponding location information is required. We can process the discreteness of all sampling sites within the range into a matrix. Each sampling site corresponds to a specific position coordinate (longitude, latitude), and the heat value of the sampling site is calculated by Gaussian fitting. The area represented by the rectangular lattice consists of longitude ($Lon_{1},\;Lon_{2}$), latitude ($Lat_{2},\;Lat_{1}$), and the matrix size is M × M. Then, the latitude and longitude corresponding to the hotspot of the row *r* and the column *c* can be calculated by Equations (7) and (8).

(7) $$lat=r\times \frac{\left(Lat_{2}-Lat_{1}\right)}{M}+Lat_{1}$$

(8) $$lon=c\times \frac{\left(Lon_{2}-Lon_{1}\right)}{M}+Lon_{1}$$

Combining the heat value with the corresponding coordinates, and then rendering the map with the corresponding chromaticity through the color table, we can further enhance the heat perception of the risk distribution. Therefore, the generation process is shown in Algorithm 1.


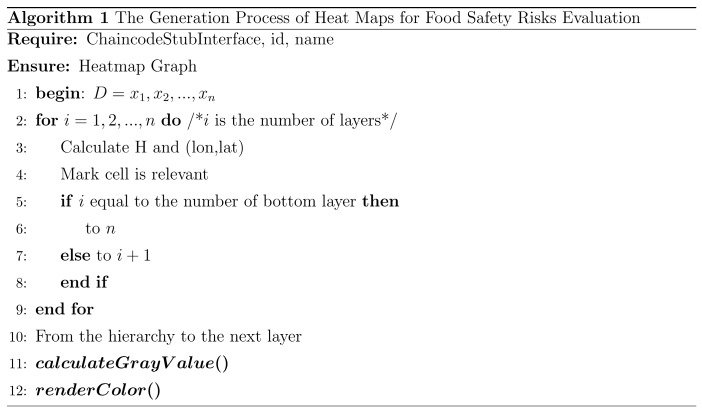


#### 4.3.2. Micro Analysis

For the risk distribution results, micro analysis is needed to discover the reasons for these risks occurring. Micro analysis is achieved through migration graphs and force-directed graphs. Migration diagrams can show relationships between regions with risks. Migration graph technology calculates, analyzes, and visualizes the data of a geographic location-based service (LBS) to dynamically, instantly, and intuitively display the trajectory and characteristics of data [35]. This technique is widely used in the analysis of movement. For example, Baidu launched a technology project called “Baidu Migration Map” during the Chinese Spring Festival Transport in 2014. It analyzed mobile phone user’s positioning information to map their tracks. This was used to observe the movement situation of China, as well as its provinces, cities, and districts in the current and earlier time periods, so as to intuitively determine the source and destination of the population. However, the migration graph can only mark the general flow direction, so the traceability of a specific product needs to be achieved through a force-directed graph.

Force-directed graphs can be used to illustrate specific flow directions, since they make relationships clear. Force-directed graphs are mainly used in the visualization of complex networks such as social networks [36]. They show the relationship between multiple nodes. Nodes are configured in two- or three-dimensional space, and the relationship between them is represented by lines. These lines are almost equal in length and as far as possible do not intersect. Both nodes and lines are subjected to forces (Coulomb repulsion, Hooke’s law, and damping attenuation also should be considered [37]), which can be calculated according to the relative positions. The motion trajectories of the nodes are determined according to the action of the forces, and their energy is continuously reduced, eventually reaching a stable and balanced state. Through predefined points, edge weights, and other information, the force-directed graph can reflect the data flow direction according to the real-time state.

In order to better understand the principle of its generation, we use physical methods as an analogy. First, the entire network can be considered as a virtual physical system. Each node of the network can be considered as a discharge particle in the system with a certain energy and, in the proposed method, represents a place (the start or end location of the unqualified products flow). The forces reflect the strength of these relationships among places. There is Coulomb repulsion between particles, which makes them repel each other. At the same time, some particles are implicated by some edges (lines), which can reflect the flows of the relationships in the method. These edges generate hooke ’s gravitational force to keep the particles at both ends of the edge. Under the continuous action of repulsion and gravity between particles, the particles are constantly displaced from the random and disordered initial state, and gradually tend to balance to form an orderly final state. At the same time, the energy of the entire physical system is also continuously consumed. After several iterations, relative displacements between the particles almost no longer occurs, and the entire system reaches a stable and balanced state. Based on this, the process of generating a force-directed graph is as follows.

- 1. The initial nodes positions are distributed randomly.
- 2. Calculate the unit displacement (edge/ line) caused by the repulsive force and gravitational forces between any two nodes in the area at each iteration.
- 3. Constantly adjust according to parameters such as the distance between nodes, the location of nodes, and the repulsive and gravitational coefficients.
- 4. Add up the unit displacements (edges/ lines) of all nodes.
- 5. Iterate n times until the desired effect is achieved.

Through the use of migration graphs and force-oriented graphs, regulators can more intuitively observe the causes of risks. Therefore, relevant rules can be formulated to reduce the occurrence of food safety incidents and ensure a food safety environment.

## 5. Experiments and Numerical Analysis

### 5.1. Experiment Platform

This research work is based on the Hyperledger Fabric v1.0 for development. The underlying environment is ubuntu16.04, Git client version 2.5.1, Node.js v8.11.2, NPM v5.6.0, Docker version 17.12.0-ce, Docker Compose-v1.8. The development languages are Python 3.6.0 and Go v1.9. LevelDB is used as the database, which is also the default database built into the Hyperledger. The front end is mainly developed through Hyper Text Markup Language (HTML), Javascript, JQuery, and Cascading Style Sheet (CSS). The background includes server deployment, information uploading to the blockchain, and information display of the blockchain. In the experiment, the CPU model of the microcomputer is Intel(R) Xeon(R) CPU e5-2603 v3@1.60ghz *6, and the memory is 8GB.

The description of the system interfaces can be divided into four parts. Part A the main interface of the system. It contains three main users of the system, which are inspectors, transporters, and users (manufacturers, processors and distributors). Here we take the transporter as an example. When the transporter completes the transportation of a batch of products, the detailed information of the products needs to be recorded and uploaded. Part B is the data upload interface, which contains detailed information of the transported products, such as name, ID, date, and so on. After the data upload operation is finished, the consensus of the nodes needs to be completed to ensure the validity of the data. Then, the valid data can be uploaded to the blockchain. Part C is the visual display interface. The upper part shows the relationship between the three roles, and the other part demonstrates the blockchain. It shows the basic information of blocks such as block number, hash value, and date. Part D shows the detailed information, which includes size, nonce, and so on. It can be used to analyze data traceability.

**Figure 4:**
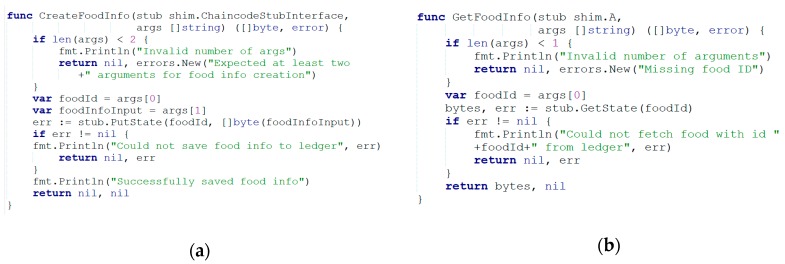
(**a**) Upload the data to the blockchain. (**b**) Retrieve the data from the blockchain.

Hyperledger Fabric uses container technology such as Docker to host the “chaincode” (i.e., smart contracts) which include the system application logic. Smart contracts typically use the Domain-Specific Language (DSL) for development. These languages have specific restrictions for blockchain, like Ethereum. It is not only not conducive to the use of ordinary users, it also causes greater difficulties for developers. One of the features of Hyperledger Fabric is that it uses general-purpose programming languages such as Java, Node.js, and Go to develop smart contracts. It greatly reduces the learning costs for developers. In addition, it is the only channel for users to interact with Hyperledger Fabric and the only tool for performing transactions on the blockchain platform [38]. Based on these, we use smart contracts to implement the operations of recording data to the blockchain and downloading data, as shown in Figure 4.

**Figure 5:**
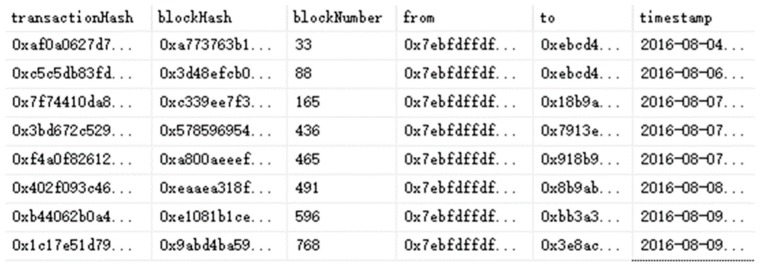
Data in the blockchain.

Since the sampling results are stored on the blockchain, we collected the sampling data for the whole year of 2016. In order to protect the privacy of the merchant, we performed address hiding and other processing on the data, and the results are shown in Figure 5.

As shown in Figure 5, using the blockchain platform to store the sampling data can meet the following security requirements: (1) Privacy protection. The merchant information stored in the block is hash encrypted (from and to). It does not disclose any private information about merchants, thus achieving privacy protection and improving security. (2) Data integrity. After the information is record into the block, it will correspond to a specific block number (blockNumber), and the block will be encrypted by the hash function to generate a unique hash value (blockHash). (3) Information security. Digital signatures are used to prove ownership, and the hash function is also used to encrypt transaction information (transactionHash). In addition, the use of digital signatures can prevent duplicate transactions. This ensures the security to a certain extent.

### 5.2. An Example of Using Aquatic Products Data

The data set used in this research is the Food Sampling Data of the full year of 2016. The data set contains 341 types of food with a total of 1,048,575 items. The detailed parameters are shown in Table 1. Here we have selected the data related to 19,578 aquatic products as the experimental data set, which includes fish, shrimps, crabs, shells, and mollusks. Among these, a total of 19,142 items were marked with qualified items. There were 436 items marked as unqualified items, question items, and non-determined items. In order to protect the privacy of the merchants, we blurred the information in the data set and processed the detection results in proportion to ensure the validity of the results. 

#### 5.2.1. Quantitative Risk Analysis Based on Heat Map

**Figure 6:**
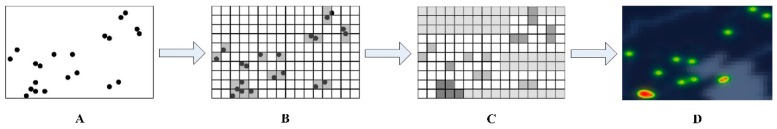
Schematic diagram of the generation process.

In order to visualize the areas where unqualified products appear, we use a heat map here. The generation results are shown in Figure 6. (A) and (B), where we first conduct grid clustering based on unqualified products data. (A) shows relatively rough clustering results, and (B) is a graph representing the results with accurate clustering. After the data is processed, the gray value, which reflects the color depth of the points in the black and white image, is superimposed and displayed as shown in (C). Then, the value is mapped using a color-band with 256 colors to finally form a colored heat map, as shown in (D).

**Figure 7:**
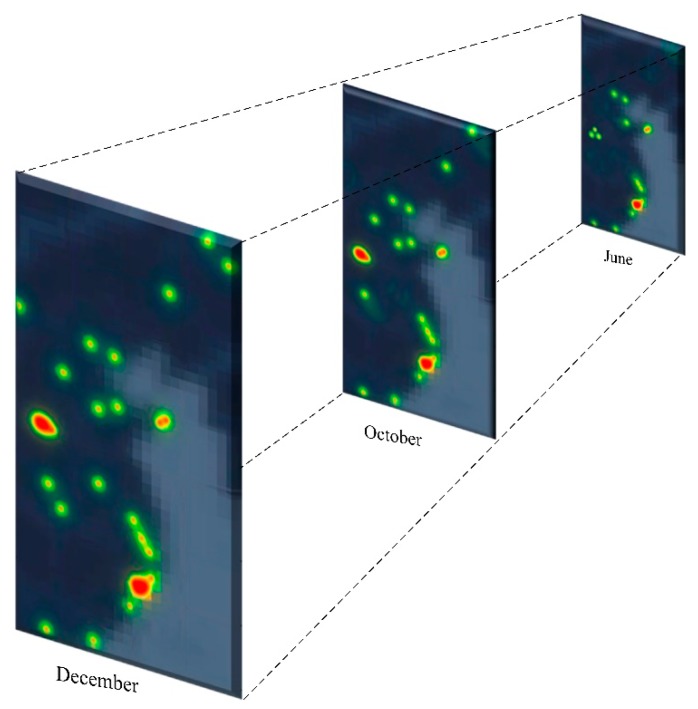
Heat maps over time.

**Figure 8:**
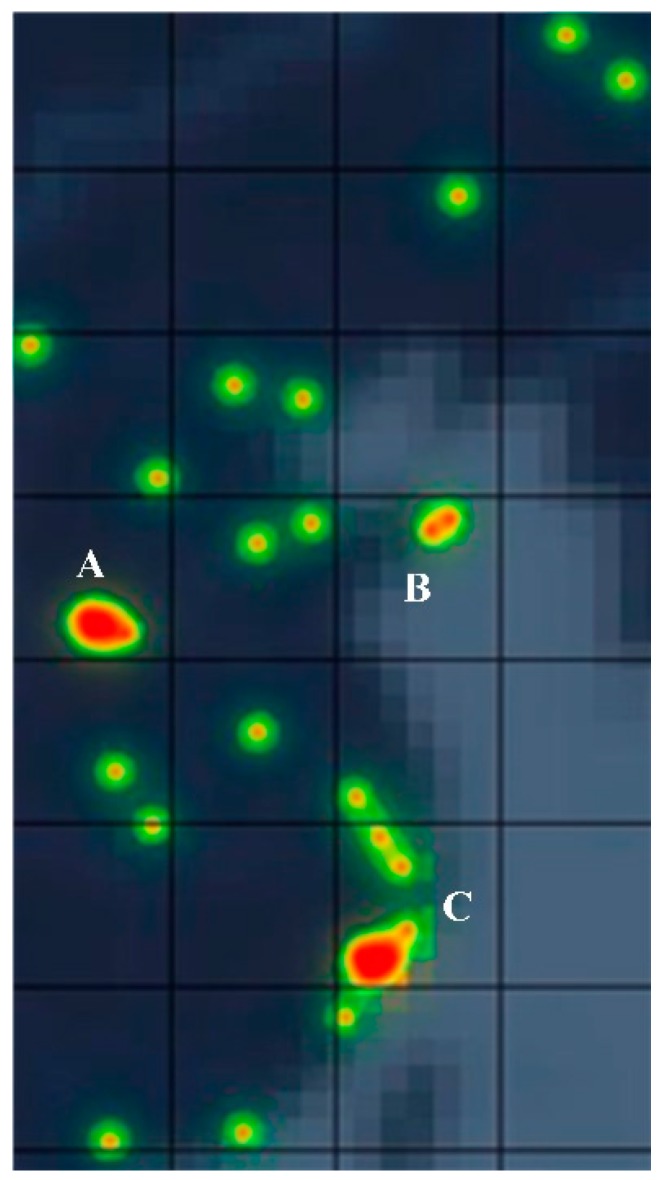
Part of the heat map.

The heat map will show different results over time. In order to measure this, we built a 3D model similar to [39,40]. Figure 7 shows the results of the heat map changes from June to December 2016. In order to better present the results, we selected the results in June, October and December. From Figure 7, it can be seen that most of the areas where incidents appear are initially near the water. Incidents are concentrated in some areas. As time goes on, incidents have gradually emerged in inland areas and some areas have a tendency to concentrate. From June to October, in particular, this trend is obvious. For the areas where incidents occur, we use the results from December for analysis (see Figure 8).

For better analysis, the pictures were divided into 28 different grids. It can be seen from Figure 8 that large red areas appear in grids A, B, and C, which indicates that a large number of unqualified items occurred in these three grids. In particular, continuous red areas appeared in grid C, which indicates that the unqualified items occurred more frequently in grid C. Therefore, we recommend finding solutions for all grids where incidents occur. For grids A, B, and C, a high degree of attention must be paid to the resolution process to reduce the risk of the incident recurring. In this way, special solutions can be formulated for different causes of problems in different places, greatly improving the efficiency of problem solving.

#### 5.2.2. Traceability Analysis Based on Migration Map and Force-Directed Graph 

**Figure 9:**
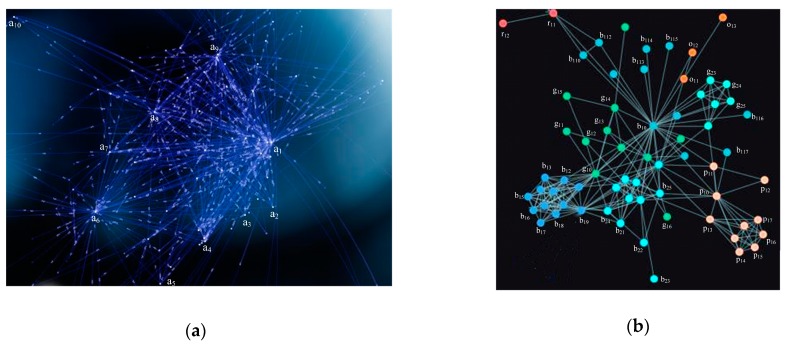
(**a**) Migration map and (**b**) force-directed graph illustrating that they can realize traceability analysis of the unqualified products.

**Figure 10:**
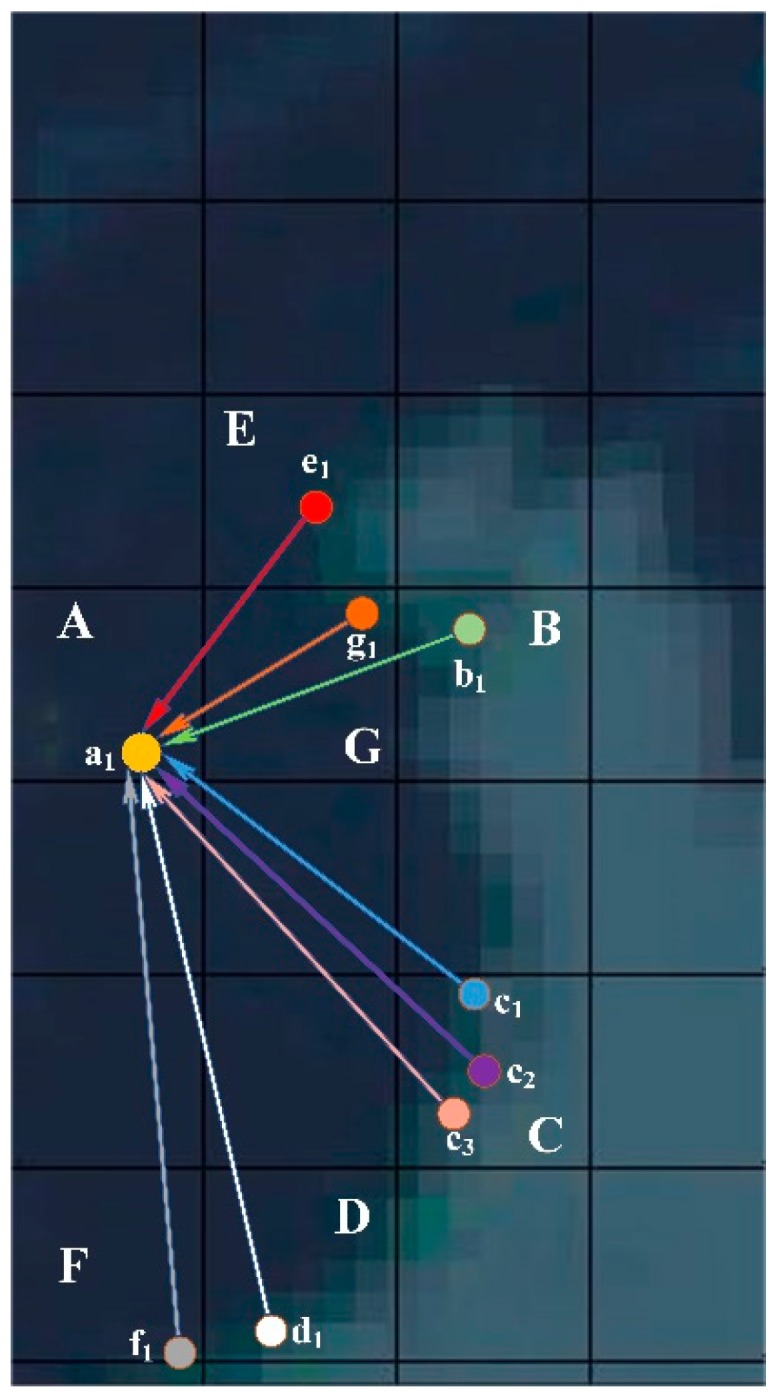
Traceability analysis graph of unqualified products.

In order to provide effective evidence to facilitate the development of solutions, we need to conduct a traceability analysis based on the above results. We select a_1_ in Grid A for further analysis. As shown in Figure 9, the migration map and force-directed graph realizes the traceability analysis of unqualified products. These product parameters are shown in Table 2. However, the results presented in Figure 9 are too complex and greatly increase the difficulty of analysis. Thus, we simplified the original image for better analysis and produced Figure 10. As can be seen from Figure 10, the unqualified products appearing in a_1_ come from eight locations in grids B, C, D, E, F, and G, which are b_1_, c_1_, c_2_, c_3_, d_1_, e_1_, f_1_, and g_1_. These results reflect the flow of unqualified products. Analyzing these flow processes can help regulators in these regions perform better management. From Table 2 and Figure 10 it can be observed that the unqualified products appear in regions near the water. The regulations in these areas can be strengthened to reduce the occurrence of unqualified products. For example, regulators need to formulate stricter regulations, such as increased frequency of spot checks on products, and increased penalties for manufacturers of unqualified products [41].

## 6. Conclusions

Most current research only uses blockchain technology to ensure the authenticity and validity of the data. This research not only proposes a method based on blockchain technology to realize the storage and management of food sampling data, but also introduced visualization methods to intuitively show risks and help the traceability analysis of food. This research can expand the current research system from the following aspects.

First, unlike the current data storage and management methods, the designed data structure can meet the needs of different roles and normalize the recording rules. In addition, the blockchain technology is used to store data. Since the blockchain features are distributed, the stored data cannot to be tampered with maliciously. Therefore, the reliability and integrity of data can be ensured. Second, most current methods for risk analysis are qualitative, that is, the risk is classified into several levels. Based on food sampling data, a quantitative analysis method has been proposed for food safety risk assessment. We have proven that this method can provide a scientific basis for management, thereby reducing the occurrence of risks and protecting people’s health. Finally, unlike many current analysis methods that only focus on the results, a holistic and detailed analysis approach has been adopted to obtain results and the reasons for their occurrence, respectively. The experimental results visualize risks through heat maps, and the traceability can be analyzed through migration and force-oriented graphs. By using visualization methods to display information, people can easily mine some important data. In practical implications, this research can help regulators (such as the Food and Drug Administration) through using these results to develop more scientific and reasonable regulatory strategies for reducing the occurrence of food safety incidents. In addition, it can also facilitate the management and control of the regions with risks.

However, there are still deficiencies that need to be improved. Blockchain technology has issues related to the speed and scalability of generating blocks. These problems will greatly reduce the efficiency of data processing. In addition, the visual analysis is also affected by the quality of the sampling data, and problems such as sampling errors can greatly affect the validity of the results. In the future, we should consider effective methods to solve these problems in order to obtain more accurate results.

## Figures and Tables

**Table 1:** An example of the dataset.

ID	Product ID	Product Name	Place of Production	Place of Sold	Food Category ID	Food Category	Substance ID	Substance Name	Result	Judgement	Date
1	93074	Razor clam	C_1_	A_2_	745	Shell	136	Tetracycli-ne	0	Qualified	1/1/2016

**Table 2:** Parameters of the unqualified products.

ID	Product ID	Product Name	Place of Production	Place of Sold	Food Category ID	Food Category	Substance ID	Substance Name	Result ^1^	Judgement	Color
1	60813	Sea crab	b_1_	a_1_	738	Crab	41	Cadmium	1.481364	Unqualified	Green
2	4708	Croaker	c_1_	a_1_	737	Fish	184	AOZ	42.804198	Unqualified	Blue
3	96064	Scylla serrata	c_2_	a_1_	738	Crab	41	Cadmium	3.7944408	Unqualified	Purple
4	93568	White shrimp	c_3_	a_1_	736	Shrimp	1027	AOZ	1.9691520	Unqualified	Pink
5	11859	Weever	d_1_	a_1_	737	Fish	1027	AMOZ	3.344956	Unqualified	White
6	9290	Turbot	e_1_	a_1_	736	Fish	182	SEM	56.294333	Unqualified	Red
7	93109	Pomfret	f_1_	a_1_	737	Fish	1027	AOZ	1.1054634	Unqualified	Gray
8	9415	Mantis Shrimp	g_1_	a_1_	736	Shrimp	123	Chloramp-henicol	0.304360	Unqualified	Orange

^1^ This refers to detection results. Due to the length limitation here, only six digits after the decimal point are retained.
